# What Do District Health Managers in Ghana Use Their Working Time for? A Case Study of Three Districts

**DOI:** 10.1371/journal.pone.0130633

**Published:** 2015-06-11

**Authors:** Marc Bonenberger, Moses Aikins, Patricia Akweongo, Xavier Bosch-Capblanch, Kaspar Wyss

**Affiliations:** 1 Swiss Centre for International Health, Swiss Tropical and Public Health Institute, Basel, Switzerland; 2 University of Basel, Basel, Switzerland; 3 School of Public Health, University of Ghana, Legon, Ghana; University of Oxford, KENYA

## Abstract

**Background:**

Ineffective district health management potentially impacts on health system performance and service delivery. However, little is known about district health managing practices and time allocation in resource-constrained health systems. Therefore, a time use study was conducted in order to understand current time use practices of district health managers in Ghana.

**Methods:**

All 21 district health managers working in three districts of the Eastern Region were included in the study and followed for a period of three months. Daily retrospective interviews about their time use were conducted, covering 1182 person-days of observation. Total time use of the sample population was assessed as well as time use stratified by managerial position. Differences of time use over time were also evaluated.

**Results:**

District health managers used most of their working time for data management (16.6%), attending workshops (12.3%), financial management (8.7%), training of staff (7.1%), drug and supply management (5.0%), and travelling (9.6%). The study found significant variations of time use across the managerial cadres as well as high weekly variations of time use impulsed mainly by a national vertical program.

**Conclusions:**

District health managers in Ghana use substantial amounts of their working time in only few activities and vertical programs greatly influence their time use. Our findings suggest that efficiency gains are possible for district health managers. However, these are unlikely to be achieved without improvements within the general health system, as inefficiencies seem to be largely caused by external factors.

## Introduction

Ineffective health management potentially impacts on health system performance and service delivery [1, 2]. Activities of health managers typically comprise areas such as planning, organizing, monitoring, supervising, directing, managing human resources, coordinating, resourcing and budgeting [3]. It is generally assumed that such managerial activities improve organizational outcomes, if managers perform them effectively [4]. For instance, a study on human resource management (HRM) showed that good human resource (HR) practices were associated with decreased patient mortality [1]. Another study also found that improved management practices contribute to lower mortality, higher institutional income, and better patient satisfaction [2].

An important factor for good management practices is effective time management. Claessens et al. [5] define time management as “behaviors that aim at achieving an effective use of time while performing certain goal-directed activities”. This definition focuses on the goal-directed activities, such as work tasks, which are carried out by making an efficient use of time. Studies have shown that improved time management practices are positively associated with job satisfaction, reduced stress, and work performance [6–8].

To our knowledge this study was the first to explore time use practices of district health managers in Africa or elsewhere. District Health Management Teams (DHMTs) in Ghana are responsible for managing all areas of health service delivery at district level. They were established following the Ghana Health Service (GHS) and Teaching Hospital Act (Act 525) in 1996, which initiated decentralization of Ghana’s health services with the subsequent creation of Regional Health Administrations (RHA) and District Health Administrations (DHA) [9, 10]. DHMTs are headed by District Directors of Health Services (DDHS), who are responsible for policy translation, implementation, coordination, monitoring and evaluation, and who provide direction in the management of health service delivery in their districts [11]. To carry out these tasks they are assisted by administrative, technical and operational managers such as health services administrators, disease control officers, and supply officers. Although district health managers are crucial at the operational level of health systems, surprisingly little is known about how these managerial cadres structure their workdays.

Time use studies are usually conducted with health workers at facility level [12–16]. As these personnel are usually centered on their workplace and are, therefore, not very mobile at work, time and motion techniques are often used as the method of choice to measure work time. This method involves the direct observation of health workers by trained observers, who record activities at short time intervals. Although regarded as the ‘gold standard’ for measuring work time of individuals [17, 18], time and motion methods are often not feasible for larger time use surveys or study groups with increased mobility, as only a limited number of individuals can be followed by each observer [18]. In such studies, self-administered timesheets or retrospective interviews are often the preferred methods to collect time use data of health workers [19–23].

This study was carried out within the framework of PERFORM, a health human resource management (HRM) intervention program, which aims at identifying ways of strengthening decentralized district management in order to improve health workforce performance in sub-Saharan Africa [24]. Although PERFORM is carried out in three African countries, this study focused only on time use practices of DHMTs in Ghana. The aim of this study was to assess current time use practices of district health managers in the three PERFORM study districts and to identify ways to improve their time use. We, therefore, conducted an explorative three-month DHMT time use study by doing daily retrospective time use interviews with all district health managers in the study districts and by using a time diary approach.

## Methods

### Study setting

The study was carried out in the Akwapim North, Upper Manya Krobo, and Kwahu West districts, which are located in the Eastern Region in Ghana. These districts were selected by PERFORM on the basis of their performance according to a baseline analysis of indicators of the 16 functioning districts in the region, which resulted into a grading of good, moderate and poor performance. Of the 11 districts that expressed interest to participate in the study we selected a well (Kwahu West), a moderately (Akwapim North), and a poorly performing district (Upper Manya Krobo).

All three DHMTs in these districts were included in the study, in which a total of 21 district health managerial staff were working during the data collection period in 2013. As shown in Table 1, six, seven and eight staff were available in the respective districts. DDHSs, public health nurses, disease control officers, health information officers, and financial officers were available in all three DHMTs. The position of the health services administrator was vacant in two DHMTs and that of the nutrition officer and supply officer each in one DHMT. The position of the human resource officer was vacant in all three DHMTs during the study period.

**Table 1:** Compositions of the study DHMTs.

	Akwapim North	Upper Manya Krobo	Kwahu West	Total
**Administrative managers**	**2**	**1**	**1**	**4**
District directors of health services	1	1	1	3
Health services administrators	1	0	0	1
**Technical managers**	**4**	**4**	**4**	**12**
Public health nurses	1	1	1	3
Disease control officers	2	1	1	4
Health information officers	1	1	1	3
Nutrition officers	0	1	1	2
**Operational managers**	**2**	**2**	**1**	**5**
Finance officers	1	1	1	3
Supply officers	1	1	0	2
Human resource officers	0	0	0	0
***Total***	***8***	***7***	***6***	***21***

Our study coincided with two nationwide immunization campaigns, which were carried out within the framework of the Expanded Program on Immunization (EPI) in order to vaccinate infants and children against polio as well as measles/rubella. In Ghana, national and sub-national immunization campaigns are regularly conducted as supplementary activities to routine immunization services to increase coverage [25]. While national polio immunization campaigns are carried out annually since 1996 [26], national measles supplementary immunization activities are implemented in four-year intervals since 2002 [27], most recently in 2013. As the district level manages and coordinates the implementation of national immunization campaigns, our study provides a unique insight on how such campaigns influence time use of district health managers as compared to their routine time use.

### Tool development

Development of the time recording tool followed two steps. First, we consulted relevant health management literature, policies and manuals in order to identify typical activities and tasks of health managers. Because the GHS currently provides job descriptions only for the DDHS, but not for other DHMT cadres, we consulted job descriptions for similar professions at hospital level, which are available for all cadres at this level. We conceived of meaningful activity categories and assigned the identified activities to these. The categories were “managing and monitoring service provision”, “human resource activities”, “management of material resources”, “financial management”, “general management activities”, “clinical activities”, “travelling” and “non-productive activities”. Secondly, we conducted individual interviews with all 21 DHMT members of Akwapim North, Upper Manya Krobo, and Kwahu West and asked respondents about missing activities, the validity of our identified activities, and the frequency in which they conducted these activities. The tool was revised after the interviews to remove irrelevant activities and activities of low frequencies, and to add missing activities frequently conducted by district health managers.

### Pilot

In order to further assess the validity of the activities for all studied DHMT positions and to identify the most suitable method for tool delivery, we conducted a two-week pilot in the three study DHMTs. We tested three methods: self-administration, face-to-face interviews and telephone interviews. Self-administration was not suitable for the study as the response rate of only about 50% was low and also because district health managers found it difficult to assign their activities to the correct activity in the time recording tool, which would have created harmonization issues in the analysis. We found that the best method for carrying out the study was through a combination of face-to-face interviews and telephone interviews: face-to-face interviews with all district health managers present in the DHAs early in the morning and telephone interviews with those staff who did not report at the office due to responsibilities outside the DHA on a certain day. Whereas interview-administration had the advantage that the interview took place in a familiar office environment and during a time respondents were less busy, telephone interviews were the only feasible way to reach study participants who were absent from the office on a given day. Accuracy of both methods was superior to self-administration as one trained person assigned all activities from the respondents in a district to the time recording tool. The tool was revised again after the pilot mainly to make activities more specific and to add or remove activities (see S1 File for the tool version used in this study). The activities measured in this study as well as their definitions are presented in Table 2.

**Table 2:** Definitions of the activities included in the study.

Activity	Definition
Planning & organizing of health services and programs	All activities related to the development, writing, and organizing of health services and health programs.
Monitoring	All monitoring activities, including preparation and post-processing of these activities, such as monitoring staff performance, work progress, and availability of drugs and supplies in health facilities.
Data management	Activities related to routine health service data from health facilities, including obtaining, collating, entering, validating, revising and analyzing of data, and subsequent reporting.
Project reports	Writing of reports not related to routine health data, such as progress reports for health projects carried out for stakeholders.
Community visits	All visits to the communities of the district, representatives of the communities or participation in community durbars.
Research	All activities related to research, including PERFORM.
HR Management	All activities related to the management of HR, such as updating of the HR database, preparation of promotions, and writing of the monthly HR report.
Supervision	Activities related to supervision of staff, including preparation and post-processing of supervisory visits.
Training	All training events carried out by DHMT staff, including preparation and post-processing of such events.
Staff durbars	Participating in or organizing of staff durbars.
Management of buildings and equipment	All activities related to the management of buildings and of medical and technical equipment, such as updating of asset registers, receiving computers or medical equipment, and activities related to maintenance, repairing and rehabilitation.
Management of drugs and supplies	All activities related to the management of drugs and supplies and their provision to health facilities. Activities include procurement, updating procurement registers, and issuing of drugs to health facilities.
Financial management	Activities related to the management of finances, such as preparing and issuing payment vouchers, budgeting, and financial reporting.
Meetings & visitors	All activities with regard to attending meetings and receiving visitors, including preparation and post-processing of such events. Activities include weekly DHMT meetings, meetings with stakeholders at all levels, unplanned emergency meetings.
Workshops	Participating in workgroups, workshops or conferences, which are organized by any agency of MoH, other sector-ministries, or third-party organizations.
Administration	All administrative activities in the office, such as phone calls, mailing, or letter writing.
Self-study	All activities intended to obtain new knowledge, but which are not related to formal training.
Clinical activities	All health promotion, prevention and curative services, such as talking on radio shows on health topics, x-ray screening, ward rounds, and attending to patients at OPD.
Travelling	All activities related to travelling, such as journeys to health facilities, villages, and venues of meetings and workshops.
Non-productive activities	All activities, which are unproductive such as waiting for an activity to start, morning devotions, and private social commitments.
Other activities	All activities, which do not belong to any of the above activities.

### Data collection

We posted one field assistant to each of the three DHAs. Two field assistants already had working experience in a DHA and were, therefore, familiar with common activities of DHMTs. All field assistants received formal training on interview techniques prior to the start of the data collection. In order to ensure consistency of the interviews we handed out a documentation guideline to all field assistants and study participants, which precisely described the activities listed in the time recording tool. All 21 district health managerial staff in the three DHMTs were included in the study and were followed over a period of three months between 1^st^ August and 31^st^ October 2013. Daily retrospective time use interviews were conducted by the three field assistants. They asked DHMTs for all their professional activities of the previous day from start of the work in the morning up to the end of the working day. Activities as well as their start and end times were recorded in time use diaries as reported. After each day, field assistants assigned the recorded activities and times to the corresponding activities in the time recording tool. Accuracy of these allocations of activities and time use was regularly checked by a field supervisor and emerging issues were discussed with field assistants, study participants, and within the core research team.

### Statistical analysis

We entered the data using Epi Info 7 and used STATA 13 (STATA Corp., College Station, TX, USA) for the statistical analysis. The data was checked for correctness and implausible values. Due to the explorative nature of the study, we applied simple descriptive statistics to analyze the data. Total work time (without breaks) was calculated by subtracting breaks from total time on duty. Means were calculated only for days on which respondents were on duty or for which information was not missing. Confidence intervals (CIs) of 95% are reported as between-subject CIs. Percentages were calculated by dividing the activities or activity categories by total work time (excluding breaks). For the analysis of the variation of weekly time use we included only activities with a greater variation, according to visual assessment, excluding all activities which remained relatively stable during the study period. Because of the non-parametric character of the data, we used the Kruskal-Wallis test to examine differences of numeric variables between groups (DHMT position or time).

### Ethics statement

This study was approved by the research commission (institutional review board) of the Swiss Tropical and Public Health Institute (#103, 25/04/12). Ethical clearance was obtained from the Ghana Health Service Ethical Review Committee (ID No.: GHS-ERC: 13/05/12). This study was an integral part of the PERFORM intervention program administered by the Liverpool School of Tropical Medicine (LSTM), and the whole intervention program thus got ethical clearance from the Research Ethics Committee of LSTM (ID No.: 12.09). Before the start of the data collection we obtained written clearance from the Eastern Regional Health Administration, Koforidua, Ghana (07/06/13). Participants gave verbal informed consent to participate in the present study, for which no particular written consent form was necessary, as they already had provided a general consent for PERFORM. Participant consent was documented in the written record by the interviewer. This consent procedure was approved by the institutional review boards mentioned above.

## Results

Overall, we recorded 1182 work days of 21 district health managerial staff over a period of 3 months. The mean time on duty (including breaks) was 9.4 hours (95% CI, 9.0 to 9.8). Because district health managerial staff spent 0.7 hours (95% CI, 0.6 to 0.8) in breaks, mean work time comprised 8.7 hours (95% CI, 8.2 to 9.1). With the working time prescribed by the GHS being 8 hours without breaks, this means that the mean overtime worked by district health managers was 0.7 hours.

**Fig 1:**
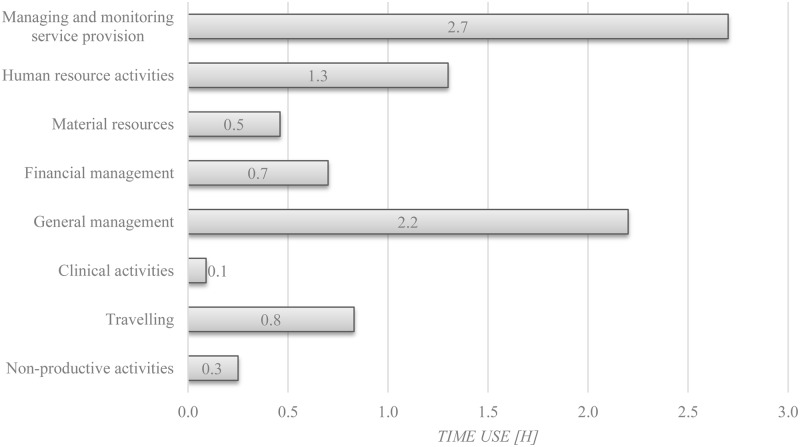
Total mean time use of district health managers, in hours. Mean working time comprised 8.7 hours. The working time prescribed by the Ghana Health Service is 8 hours.

Fig 1 shows the distribution of total mean time use of district health managers in the eight major activity categories in hours. The highest mean time was used in managing and monitoring service provision, for which managers spent 2.7 hours. General management activities were with 2.2 hours the activity group with the second highest time use, followed by human resource activities with 1.3 daily hours. In comparison, mean time use for financial management (0.7 hours) and management of material resources (0.5 hours) were considerably lower. Clinical activities were conducted only 0.1 hours in the mean. Travelling accounted for 0.8 hours and non-productive activities (excluding breaks) for 0.3 hours of the mean working time.

Table 3 presents the time use pattern for all activities carried out by district health managers during the three months study period in minutes and in percentage of total time use. District health managers allocated with 16.6% (86 minutes) the highest proportion of their working time to data management, followed by attending workshops (12.3%, 64 minutes), monitoring (9.8%, 51 minutes), and attending meetings or receiving visitors (8.5%, 44 minutes). Although human resource activities was the activity group with the third-highest proportion of time use, activities in this group were distributed unevenly, with the highest share allocated to training (7.1%, 37 minutes) and supervision (6.6%, 34 minutes) of staff, but with a low share allocated to HRM (1.3%, 7 minutes) and staff durbars (0.6%, 3 minutes). A similar pattern was observed in the material resources activity group, as managers spent most of their mean time in managing drugs and supplies (5%, 26 minutes), but allocated only a fraction of their time to managing buildings and equipment (0.2%, 1 minute). Other activities with low proportions of mean working time were community visits (0.2%, 1 minute), research (0.6%, 3 minutes), and clinical activities (1.0%, 5 minutes). District health managers spent 9.6% (50 minutes) of their mean working time on travelling and 2.9% (15 min) of their activities were non-productive.

**Table 3:** Activities of district health managers and mean time use, in minutes and percentage.

Activity	mean time [min] (95% CI)^a^	Percentage of total time use
**Managing and monitoring service provision**	**162 (121–203)**	**31.2%**
Planning & organizing	19 (14–24)	3.7%
Monitoring	51 (40–61)	9.8%
Data management	86 (51–122)	16.6%
Project reports	3 (1–6)	0.6%
Community visits	1 (0–1)	0.2%
Research	3 (1–4)	0.6%
**Human resource activities**	**81 (54–108)**	**15.6%**
HR Management	7 (0–13)	1.3%
Supervision	34 (20–49)	6.6%
Training	37 (20–53)	7.1%
Staff durbars	3 (2–4)	0.6%
**Material resources**	**28 (3–52)**	**5.4%**
Buildings & equipment	1 (0–2)	0.2%
Drugs & supplies	26 (2–50)	5.0%
**Financial management**	**45 (3–87)**	**8.7%**
**General management activities**	**132 (100–165)**	**25.4%**
Meetings & visitors	44 (35–52)	8.5%
Workshops	64 (47–82)	12.3%
Administration	18 (6–30)	3.5%
Self-study	6 (1–11)	1.2%
**Clinical activities**	**5 (1–10)**	**1.0%**
**Travelling**	**50 (43–57)**	**9.6%**
**Non-productive activities**	**15 (10–20)**	**2.9%**
**Other activities**	**1 (0–1)**	**0.2%**

^a^reported as between-subject CIs

**Fig 2:**
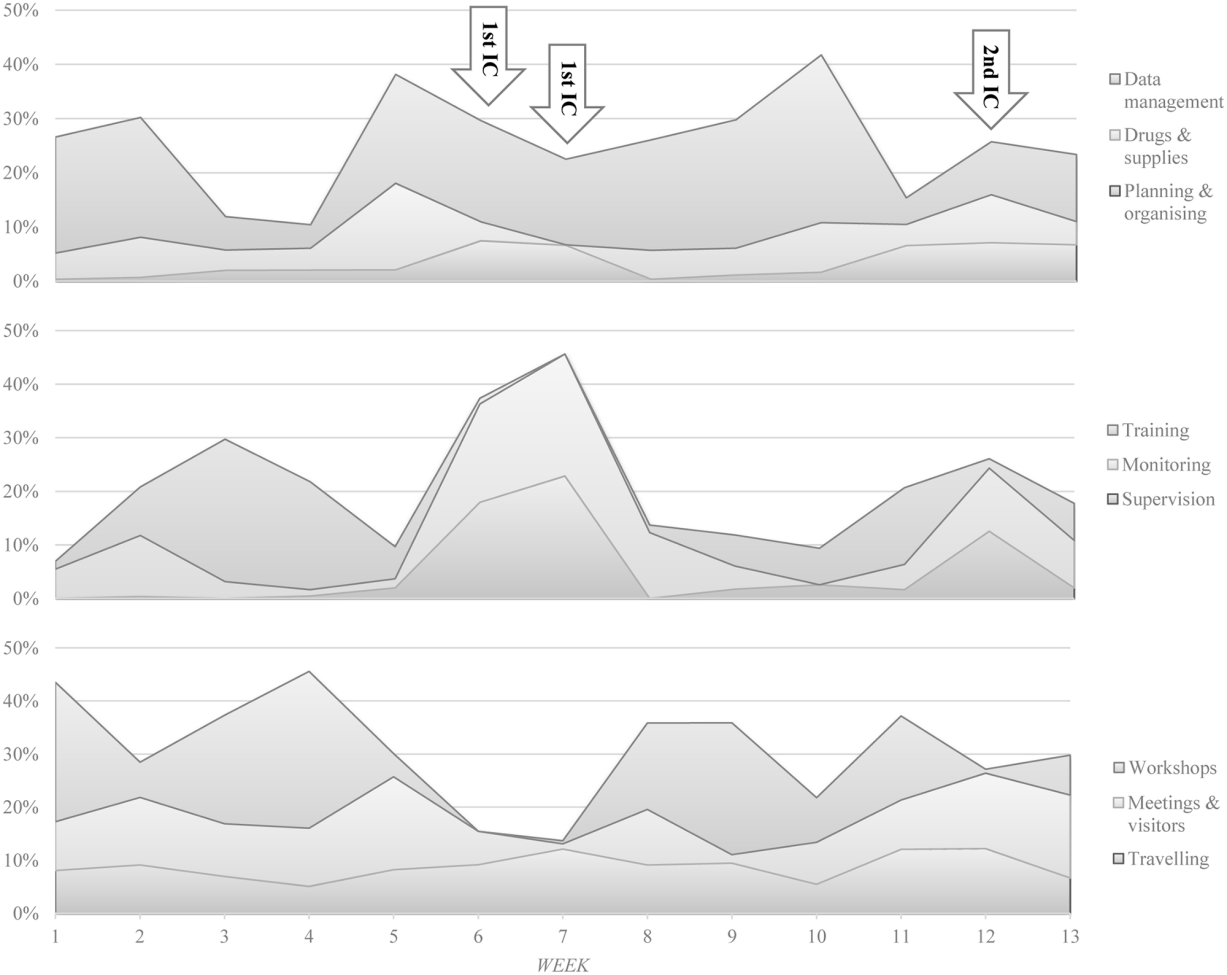
Weekly mean time use of district health managers over the three month study period, in percentage. 1^st^ IC: First immunization campaign; 2^nd^ IC: Second immunization campaign.

Fig 2 shows the variations of weekly mean time use of all study DHMTs in nine major activities of district health managers over the three months study period in percentage. Attending workshops and training of staff were dominant activities before and after the two national immunization campaigns, which were carried out during weeks 6 and 7, and week 12 of the study, but were both minor activities during these campaigns. Planning and organizing was mainly conducted shortly before and during the immunization campaigns. Also supervision was mainly conducted during these campaigns, where the activity resembled the proportion of time use in monitoring. This was because district health managers conducted supervision and monitoring simultaneously at outreach points during the campaigns and we, therefore, allocated their time use evenly to both activities during data entry. Monitoring was also conducted outside the campaigns, but the proportion of time use was highest during the first immunization campaign. Although management of drugs and supplies was a frequent activity with a distribution of 5% of total mean time use, this proportion increased shortly before or during the immunization campaigns and reached a peak with 16.0% of mean time use in week 5 during which district health managers distributed vaccines to health facilities at sub-district level. Data management was a dominant activity most of the time, as district health managers either managed routine data of the facilities at sub-district level or vaccination data from the two immunization campaigns. The proportion of this activity thereby reached with 30.9% of mean work time a peak in week 10. Attending meetings and receiving visitors, and travelling were also regular activities of district health managers, but their proportions usually increased shortly before and during the immunization campaigns.

Table 4 presents the time use pattern for the different DHMT staff categories as established during the three months study period in percentage. Most of the differences in time use between the managing cadres were found to be statistically significant. The DDHSs were the cadre with the highest percentage of working time spent on meetings and visitors (15.6%), and attending workshops (23.2%). Although all DDHSs had a clinical background, clinical activities demanded only 3.5% of their time. HRM was mainly conducted by the health services administrators in this study, to which 13.1% of the working time was allocated. Public health nurses, disease control officers, health information officers, and nutrition officers allocated a high amount of their mean working time to data management, which even reached 29.1% for the public health nurses. Nutrition officers were the cadres with the highest proportion of their working time spent in training of staff (22.8%), as they were not only engaged in training events for the two national immunization campaigns, but they also conducted training for a program on community-based management of acute childhood malnutrition (CMAM), which was initiated during the study period. Travelling was an activity conducted by all district health managers in which they spent a considerable amount of their working time with a range between 5.9% (nutrition officers) and 13.1% (supply officers). Although differences in time use were not significant for non-productive activities, these were lower than 5% of their working time for most cadres.

**Table 4:** Percentage time use across different types of district health managerial cadres by type of activity.

Activity	DDHS	PHN	DCO	HIO	NO	ADM	ACC	SO	*P* ^a^
Planning & organizing	2.8%	4.0%	5.7%	3.0%	2.7%	8.1%	0.9%	1.4%	0.003*
Monitoring	6.2%	11.2%	10.1%	11.2%	6.1%	7.9%	4.7%	12.3%	0.005*
Data management	4.2%	29.1%	28.6%	28.3%	21.7%	2.5%	0.5%	2.0%	< 0.001**
HR Management	0.0%	1.1%	0.0%	2.1%	0.4%	13.1%	0.4%	0.0%	< 0.001**
Supervision	6.8%	6.8%	5.4%	6.1%	4.1%	7.9%	1.4%	7.2%	0.061
Training of staff	7.4%	6.2%	9.0%	6.4%	22.8%	2.1%	2.1%	0.5%	< 0.001**
Drug & supply management	0.7%	2.8%	4.1%	1.2%	0.8%	0.4%	1.0%	46.8%	< 0.001**
Financial management	0.5%	0.7%	2.1%	0.2%	1.8%	1.3%	47.0%	0.1%	< 0.001**
Meetings & visitors	15.6%	10.0%	7.4%	9.2%	5.8%	10.6%	8.7%	4.6%	< 0.001**
Attending workshops	23.2%	11.3%	12.8%	13.7%	13.8%	11.7%	6.2%	1.6%	0.002*
Administration	14.6%	0.8%	1.2%	1.6%	0.3%	16.8%	1.0%	0.0%	< 0.001**
Clinical activities	3.5%	2.0%	0.9%	0.3%	1.5%	0.3%	0.0%	0.1%	0.493
Travelling	10.9%	8.0%	8.1%	8.8%	5.9%	8.2%	10.1%	13.1%	< 0.001**
Non-productive activities	1.7%	3.4%	2.3%	1.9%	2.8%	3.7%	3.1%	6.5%	0.076

^a^Kruskal-Wallis test;

*P < 0.01;

**P < 0.001.

DDHS: District Directors of Health Services; PHN: Public Health Nurses; DCO: Disease Control Officers; HIO: Health Information Officers;

NO: Nutrition Officers; ADM: Health Services Administrators; ACC: Accountants; SO: Supply Officers

## Discussion

This study shows that district health managers in Ghana use substantial amounts of their working time in only few activities, and that a vertical immunization program and its specific activities greatly influenced their time use. Data management is definitely the activity with the highest proportion of time use relative to mean working time, although it is primarily a major activity for technical managers. Other activities of district health managers with higher proportions of time use are monitoring, supervision, training of staff, financial management, meetings and visitors, workshops, and travelling. We have shown that time use of these activities varies substantially across different managerial cadres and over time.

This study had limitations. Using retrospective interviews instead of direct observations of activities are a source of social desirability bias. This has been shown by Bratt et al. [17], who compared four approaches for measuring time use and who found that respondents overstated productive time and underreported non-productive time in time use interviews, as compared to direct observation of the time and motion method. Underreporting of non-productive activities could have also occurred in our study as these were with around 3% of mean time use rather low. Sonnenberg et al. [20] stressed that survey methods for measuring time use bear the risk of over- and underestimations of time use, especially when respondents are asked to estimate the average time spent on activities. We have minimized this risk by doing repeated daily retrospective interviews, and, therefore, did not ask our respondents for their average time use, but to report actual time use of their daily activities. Compared to direct observation, self-reporting methods for measuring time use are also prone to recall bias, in a way that activities of short duration, such as short phone calls, signing of a letter, or short conversations with colleagues cannot be accurately captured, as respondents tend to underreport these activities during interviews [20, 28]. This is especially the case, when short activities are conducted simultaneously to activities with a longer duration [29]. We addressed this problem by probing for short activities during time use interviews, although statements for time use in such activities were likely to be inaccurate estimations, as these were often distributed randomly throughout the day. However, comparative research suggests that bias from self-reported methods for collecting time use data are not necessarily greater than the observer-induced bias of the time and motion method [28].

Although not a managing duty, many of the different district health managerial cadres are also involved in clinical activities. The majority of the clinical activities recorded in our study are with regard to administering drugs to clients, and health promotion activities such as talking in radio programs on health issues. For those managers with a medical or nursing background such as the DDHSs and public health nurses, clinical work can also involve attending to in and out-patients, and—in the case of medical doctors—surgery. However, in contrast to facility health managers who often continue working in their health professions beside their managing duties [30], this study has shown that district health managers are first and foremost managers, who may sometimes also engage in clinical work.

According to results we presented in a previous study, health workers in Ghana were dissatisfied with their work environment, but rather satisfied with in-service training and supervision [31]. Dissatisfaction with the work environment was mainly related to the lack of essential medical equipment and the condition of workplaces in health facilities. Agyepong et al. [32] pointed out a decade ago that this lack was not a problem of absolute unavailability of resources, but rather one of inadequate management and administration. Our current findings suggest that this problem still persists, as district health managers severely neglect the management of buildings and equipment and use only a fraction of their working time in such activities. In contrast, the higher levels of health workers’ job satisfaction with in-service training and supervision could be a result of district health managers allocating greater proportions of their working time to these activities. This further adds to the notion that good management practices of district health managers can influence job satisfaction of health workers, thereby improving staff retention [31, 33, 34].

Travelling is an activity in which district health managers spend a substantial amount of their working time. This finding indicates the high mobility of these cadres, as they work at the interface between the strategic and the operational levels of the health system requiring them to collaborate with all levels from the national to the sub-district level [35, 36]. In our study, district health managers frequently travelled to the Ministry of Health and other ministries in Ghana’s capital Accra for information delivery and to the RHA in Koforidua to exchange or deliver information, and to attend workshops and meetings. At the district and sub-district levels frequent travelling is mandatory in order to provide on-site services to health facilities such as supervision and monitoring and to liaise with other stakeholders of the district health system.

The high percentage of time district health managers use for data management raises the question whether the generated information is effectively used for priority setting, daily management and decision-making. Since 2012, Ghana uses a web-based District Health Information Management System (DHIMS), which was introduced to improve the quality of the data by shortening the data acquisition process [37]. Although empirical research suggests that DHIMS data is relatively accurate and reliable for use [38], no research has been published to date on how district health managers in Ghana actually use this data. However, research conducted by PERFORM suggests that the DHIMS is used for problem identification and decision-making at district level, as, for instance, district health managers identified low immunisation rates through DHIMS as being a problem and, in consequence, initiated various actions to increase coverage.

Our results suggest that time use practices of district health managers in Ghana are greatly influenced by vertical programs. Although we acknowledge the positive impacts of vertical immunization programs on population health and health systems in general [39, 40], it was also pointed out that vertical programs in low- and middle-income countries tend to absorb human resource- as well as material resource capacities during campaigns thereby affecting the delivery of mainstream health services [41, 42]. This is also indicated by our data, as vertical programs limit the ability of district health managers to adequately respond to the needs of the district health systems, as the entire capacities of the study DHAs were allocated to the immunization campaigns while these were carried out. Given that this study coincided with the annual polio immunization schedule and the four-year measles/rubella immunization schedule our findings need to be understood in the light of these two vaccination campaigns, although activities related to other vertical programs (e.g. national malaria control program) may request similar time allocation patterns during the course of the year.

The high amounts of time used in only few activities by at the same time neglecting other activities suggest that district health managers may not practice efficient time management. However, inefficient time use practices frequently have external causes. For instance, data management often demand high proportions of working time, because district health managers are receiving routine data from health facilities in the sub-districts, requiring them to wait in their offices until all facility heads deliver their data during which they usually only conduct minor activities such as email writing and phone calls. As the regional and national levels do not have personnel that coordinate workshops it frequently happens that district health managers spend several days on different workshops, thereby neglecting their district work schedules. The neglect of certain important management activities such as managing of human and material resources is due mainly to the fact that managerial cadres responsible for these activities, namely human resource officers and health services administrators, are unavailable or not available in all the study districts. As Ghana does not currently have a nationwide electronic human resource management system (HRMS), for district health managers managing HR, travelling demand a high proportion of their working time, because they frequently need to travel to the capital Accra in order to effect changes in the Integrated Personnel and Payroll Database (IPPD) for district staff such as salaries and promotions.

We anticipate that there is a potential for efficiency gains in time allocation for district health managers in Ghana, especially with regard to data management and attending workshops, each contributing over 10% to total mean time use, but reaching up to around 30% in weekly variations. For instance, given that reporting of routine health data is paper-based at sub-district level, it is not surprising that district health managers spend much time with receiving, collating, and entering this data. Extending the DHIMS to the sub-district level could improve efficiency in time use. However, this would entail substantial financial investments in computer equipment and training, and might not be feasible for remote areas with limited Internet access. The continuous education system of the GHS currently seems to be extremely fragmented and not structured, as was exemplified through the high frequency of workshops organized by a variety of organizations and which were unevenly distributed over the study period. Better coordination of workshops at the national and regional levels could result in improved time allocation at district level. Except for the DDHS, job descriptions are currently not available for DHMT cadres in the GHS. These should be developed and widely distributed within the GHS so to ensure that all district health managerial cadres are fully aware of their work tasks. This would also allow the identification of training needs of individual DHMT members, which could lead to efficiency gains in the long run [43]. The introduction of an electronic HRMS, which is currently in pilot phase in the GHS, may also result in efficiency gains.

This study has shown that district health managers use a high share of their working time for activities related to vertical programs and that these encourage the supervision of health service delivery at peripheral level. It would be interesting to study whether such visits also allow simultaneous activities, such as supportive supervision for health service areas not related to vertical programs while carrying these out. Also the perceptions of the district health managerial workforce on the causes of inefficient time use and on possible means to improve efficiency have not been the focus of this study. We will address such research questions in a forthcoming manuscript on DHMT efficiency.

In conclusion, our findings suggest that efficiency gains in time use are possible for district health managers. However, these are unlikely to be achieved without improvements within the general health system, as inefficiencies seem to be largely caused by external factors.

## Supporting Information

S1 File Time use data collection tool. (PDF)
