# Supplementary material for: What Do District Health Managers in Ghana Use Their Working Time for? A Case Study of Three Districts
Source: PLoS One. 2015 Jun 11;10(6):e0130633. doi: 10.1371/journal.pone.0130633 (PMC4465977; doi:10.1371/journal.pone.0130633)
Supplement: S1 File — (PDF) [file pone.0130633.s001.pdf]

PERFORM
IMPROVING HEALTH WORKFORCE PERFORMANCE

Time-use study 2013

**District:**	**Job title:**						
Month							
Date:							
**Managing and monitoring service provision**	**Please note hours and minutes spent for activities such as: 1:15, for 1 hour and 15 minutes**						
Planning health activities/services/programmes							
Organising and coordinating health activities/services/programmes							
Health service monitoring and evaluation							
Health data management and reporting (incl. HMIS)							
Writing project reports							
Community visits and community durbars							
Research/study activities (including PERFORM)							
**Human resource**							
Management of HR (including reporting for HR system)							
Training and supervision of staffs<br>(including preparation and organisation of such events)							
Staff durbars							
**Material resources**							
Management of buildings, and of medical and technical equipment<br>(incl. procurement, maintenance, repairing and rehabilitation)							
Drugstore management and provision of drugs to health facilities							
**Financing**							
Management of finances and financial reporting							
Preparation of insurance claims							
**General management activities**							
DHMT/Committee meeting							
Other *planned* meetings and appointments<br>(including preparation and organisation of meetings)							
Unplanned/emergency meeting							
Receiving unannounced visitors<br>(such as health staff, clients, etc.)							
Participation in government workgroup, workshop, or conference							

PERFORM
IMPROVING HEALTH WORKFORCE PERFORMANCE

Time-use study 2013

Month:							
Date:							
Participation in third party (donor, NGO, etc.) workgroup, workshop,or conference							
Phone calls, mailing or letter writing/delivering							
Self-study							
**Clinical activities**							
Delivery of health promotion and prevention services							
Provision of clinical/curative services							
**Other activities not mentionend above (please specify)**							
____________________							
____________________							
____________________							
**Non-productive activities**							
Waiting for the activity to start							
Travelling							
No activity due to electricity breakdown							
Break							
Devotion							
Private social commitment							
**Total day:**	*Mo*	*Tue*	*Wed*	*Thu*	*Fr*	*Sa*	*Su*
**morning: from**							
**to**							
**afternoon: from**							
**to**							
**Total hours worked**							
**Absence in days (tick)**							
**on leave:**							
**sick leave:**							
**public holiday:**							
